# The use of a borderline zone for the interpretation of interferon-gamma release assay results for serial screening of healthcare workers

**DOI:** 10.1371/journal.pone.0235254

**Published:** 2020-06-30

**Authors:** Jae Hyeon Park, Namhee Kim, Hyunwoong Park, Taek Soo Kim, Sang-Won Park, Eun Youn Roh, Jong Hyun Yoon, Sue Shin

**Affiliations:** 1 Department of Laboratory Medicine, Seoul National University College of Medicine, Seoul, South Korea; 2 Department of Laboratory Medicine, Seoul National University Hospital, Seoul, South Korea; 3 Department of Laboratory Medicine, Seoul National University Boramae Medical Center, Seoul, South Korea; 4 Department of Internal Medicine, Seoul National University College of Medicine, Seoul, South Korea; 5 Department of Internal Medicine, Seoul National University Boramae Medical Center, Seoul, South Korea; The University of Georgia, UNITED STATES

## Abstract

**Objective:**

An interferon-gamma release assay (IGRA) is used to screen for latent tuberculosis infection (LTBI). Among IGRAs, the QuantiFERON-TB Gold In-Tube (QFT-GIT) results are highly variable, so the borderline zone has been proposed to reduce unnecessary LTBI treatment. The aim of this study was to examine the proportion of the borderline zone of QFT-GIT in healthcare workers’ (HCWs) serial IGRA and to retrospectively identify the utility of predicting tuberculosis (TB) in a moderate TB incidence setting.

**Methods:**

The participants were HCWs who had undergone serial LTBI screening between June 2013 and June 2018. IGRA-positive HCWs underwent examinations that included low-dose computed tomography (LDCT) and TB culture, if necessary. Applying the borderline zone (0.2-<0.7 IU/mL), the results were classified as definite negative, borderline negative, borderline positive and definite positive.

**Results:**

Through the follow-up of 477 HCWs, 441 (92.5%) invariant, 30 (6.3%) conversion, 2 (0.4%) reversion and 5 (1.0%) indeterminate results were observed with the manufacturer’s cutoff. Applying the borderline zone, 419 (87.8%) invariant, 22 (4.6%) conversion, 1 (0.2%) reversion and 36 (7.5%) decision pending, including 5 (1.0%) indeterminate results, were observed. At the time of screening, five TB cases were identified. Chest X-ray (CXR) identified one TB case, and LDCT identified four additional TB cases. After one year, two TB cases were diagnosed, and their screening QFT-GIT results were definite positive and borderline negative. In the Cochran-Armitage trend test, the greater the maximum difference in the QFT-GIT grade with the borderline zone was, the higher the probability of developing TB (*P*-value <0.001).

**Conclusion:**

The application of the borderline zone lowered the conversion rate but increased the decision pending rate. Introducing the borderline zone requires a careful approach, and a thorough examination needs to be performed to rule out TB in converters. HCWs with borderline QFT-GIT results also need close observation.

## Introduction

The burden of tuberculosis (TB) remains high, and it is estimated that there were 10 million cases of TB, with 1.6 million deaths, in 2017 [1]. In 2016, the incidence and mortality rates of TB in South Korea were 77 and 5.2 per 100,000 population, respectively [2]. Though the rates have decreased significantly, South Korea still has the highest incidence among the Organization for Economic Cooperation and Development (OECD) member countries. TB infection by the activation of latent TB infection (LTBI) in postnatal care centers, schools, and hospitals has become a social problem. South Korea has intensified its TB policy, including its LTBI control programs, since 2013 [2]. The management of LTBI for healthcare workers (HCWs) is becoming important because they are likely to come into contact with TB patients and immunocompromised individuals. The Tuberculosis Prevention Act, which was amended in 2016, requires the preplacement examination of latent TB in HCWs and annual screening of latent TB for high-risk HCWs.

Recently, the United States Centers for Disease Control and Prevention (CDC) updated the TB screening recommendations for HCWs [3]. Serial screening and testing for HCWs without LTBI is not routinely recommended, but it can be considered for selected HCW groups [3]. The Korean guidelines for TB, which were revised in 2017, recommend that the tuberculin skin test (TST) or interferon-gamma release assay (IGRA) be conducted for the examination of LTBI in medical institutions at their discretion [4]. Similar to the recommendations of the CDC, serial screening is performed based on each employee’s TB risk, but how to interpret the conversion is unclear [3, 4]. The IGRA is a convenient test and has higher specificity than TST, but it requires careful interpretation due to high variability. In particular, the QuantiFERON-TB Gold In-Tube (QFT-GIT; Qiagen, Hilden, Germany) test has not yet established guidelines on how to interpret results during serial testing [5, 6]. For serial QFT-GIT, the conversion rate and the reversion rate have been reported to be 4.4–6.1% and 20–60%, respectively, in low-incidence settings; however, there are inconsistent reports in moderate- to high-incidence settings [5].

The QFT-GIT results are highly variable near the diagnostic threshold; therefore, the application of the borderline zone has shown lower conversion and reversion rates than the manufacturer’s cutoff [7–12]. This approach can reduce the unnecessary treatment of LTBI, but there is a possibility of missing LTBI cases [7]. Moses et al. examined the effect of different borderline cutoffs by microsimulation for ten years using the Markov model [13]. They reported a decrease in false positives as the cutoff increased, but actual infections were missed [13]. In addition, there are few reports on how to interpret the QFT-GIT results in the borderline zone and its long-term consequences in moderate- to high-incidence settings.

The purpose of this study was to examine the proportion of a borderline zone of QFT-GIT in HCWs’ serial IGRA and to retrospectively identify the utility of predicting TB in a moderate TB incidence setting.

## Materials and methods

### Study population

We analyzed the QFT-GIT results of HCWs at Boramae Medical Center, who had undergone LTBI screening, spanning 6 years from June 2013 to June 2018. We retrospectively reviewed the medical and personnel records of those who were followed up after the screening. The risk of TB exposure of HCWs was assessed based on the Korean guidelines for TB. The high-risk group includes HCWs who screened, treated and diagnosed TB patients; the intermediate-risk group includes HCWs who came into contact with patients who are at high risk of severe TB; and the low-risk group includes other HCWs.

According to the Korean guidelines for TB, the screening consisted of chest X-ray and IGRA. And the screening is terminated for most HCWs if the IGRA is positive, but some HCWs have follow-up tests based on their preference. If the IGRA is positive, they recommended visiting an infectious disease doctor and undergoing examinations with low-dose computed tomography (LDCT) and/or sputum culture for TB. HCWs diagnosed with LTBI were offered 9-month isoniazid (9H), 4-month rifampin (4R), 3-month isoniazid/rifampin (3HR) or no treatment. HCWs with TB were treated with a standard regimen of 2 months of isoniazid (INH), rifampin (RIF), ethambutol (EMB), and pyrazinamide (PZA), followed by 4 months of INH, RIF, and EMB (2HREZ/4HR[E]). This study was approved by the Institutional Review Board (IRB) of Seoul National University Boramae Medical Center (IRB No. 10-2018-74). The IRB waived the need for consent from the participant, as the study was retrospective and the data was de-identified before analysis.

### QuantiFERON-TB Gold In-Tube test

As part of the occupational LTBI screening, QFT-GIT tests were performed by the Green Cross Labs (Yongin-si, South Korea), the Korean National Tuberculosis Association (Seoul, South Korea) and the Institute of Health and Environment (Seoul, South Korea). The Green Cross Labs conducted most of the tests. Each laboratory tested QFT-GIT in accordance with the manufacturer’s instructions.

### Definition of the manufacturer’s cutoff and new cutoff with the borderline zone

The interpretation of the QFT-GIT test using the manufacturer’s cutoff is dichotomous. The test is considered positive when INF-γ is ≥ 0.35 IU/mL after correction for the negative control. The range proposed for the borderline zone varies from study to study [7, 9, 11, 12, 14–16], but 0.2 to <0.7 IU/mL was used in this study as European studies [7, 9, 12, 15]. Uzorka et al. analyzed QFT-GIT results by dividing them into borderline (0.15–35 IU/mL) and low-positive (0.35–0.70 IU/mL) [16]. Therefore, the cutoff criterion of 0.35 IU/mL was maintained, dividing the borderline zone into a borderline-negative zone and a borderline-positive zone. Therefore, a QFT result of < 0.2 IU/mL was considered definite negative, a result of 0.2 to < 0.35 IU/mL was considered borderline negative, a result of 0.35 to < 0.70 IU/mL was considered borderline positive, and a result of ≥ 0.7 IU/mL was considered definite positive. Indeterminate results are defined as a test where the negative control was ≥ 8 IU/mL or the positive control ≤ 0.5 IU/mL according to the manufacturer's recommendations.

### Definition of conversion and reversion

With the manufacturer’s cutoff, conversion was defined as negative (< 0.35 IU/mL) → positive (≥ 0.35 IU/mL), and reversion was defined as positive (≥ 0.35 IU/mL) → negative (< 0.35 IU/mL). Because introducing the borderline zone determined the value of that zone as “decision pending”, conversion was defined as definite negative (< 0.2 IU/mL) or borderline zone (0.2 to < 0.7 IU/mL) → definite positive (≥ 0.70 IU/mL), and reversion was defined as definite positive (≥ 0.70 IU/mL) → definite negative (< 0.20 IU/mL). The changes that did not meet these criteria were 1) borderline zone → definite negative; 2) definite negative or definite positive → borderline zone; and 3) indeterminate results, and these changes were defined as “decision pending”.

### Statistical analysis

Descriptive characteristics are provided as frequencies for categorical variables and as the median and interquartile range (IQR) for continuous variables. The Mann-Kendall trend test was used to compare the conversion rate according to the follow-up order. After introducing the borderline zone, TB incidence according to the difference in the interpretation grade of the QFT-GIT was analyzed by the Cochran-Armitage trend test. Statistical significance was defined as a *P*-value < 0.05. Statistical analyses were conducted using R software version 3.6.1 (R Project for Statistical Computing, Vienna, Austria).

## Results

### Initial QFT-GIT results of HCWs

**Fig 1:**
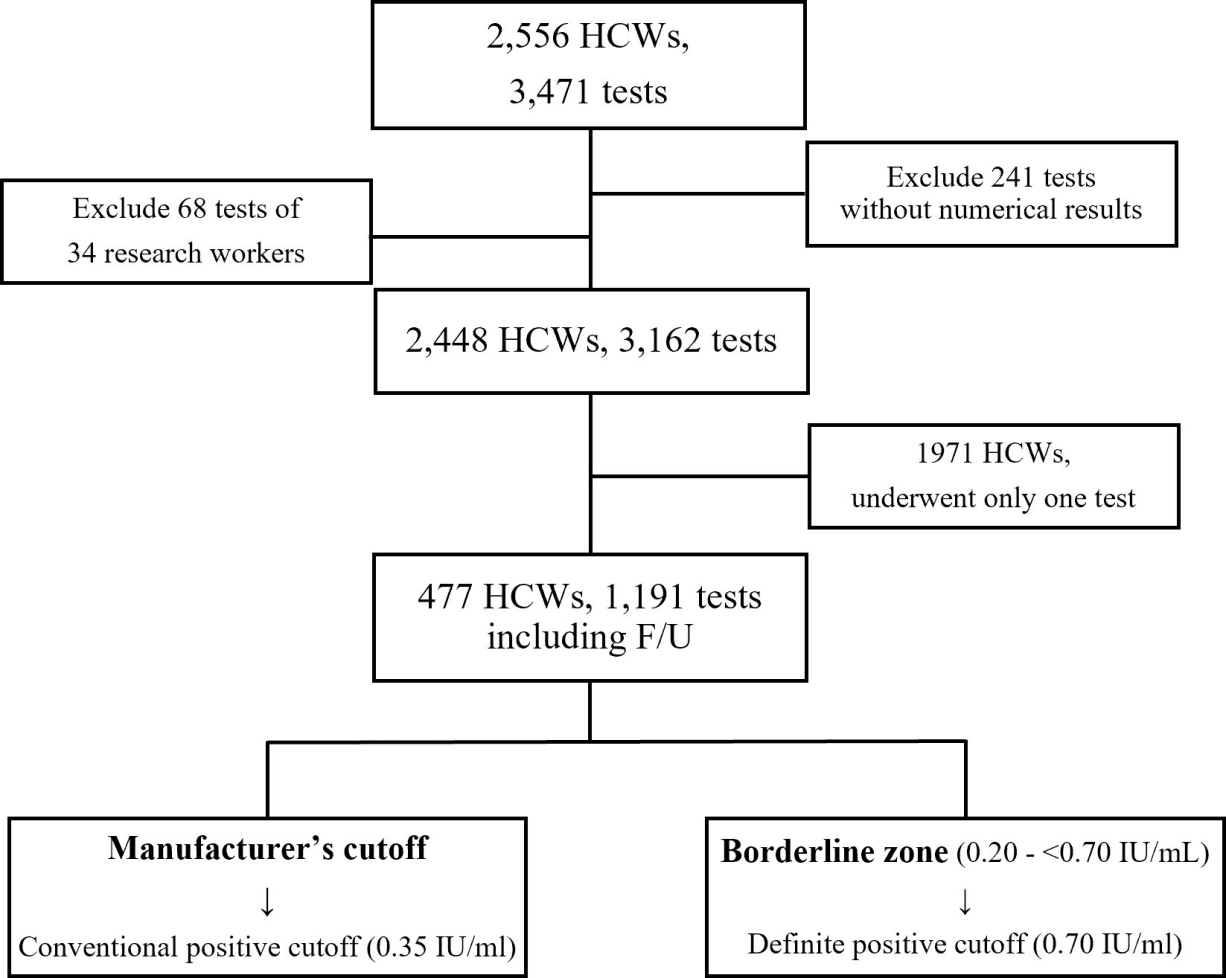
Flow chart of the study population. Abbreviations: HCW, healthcare worker; F/U, follow-up.

A total of 3,162 tests and 2,448 HCWs were included, excluding 241 tests without numerical results and 68 tests of researchers working in separate locations (Fig 1). Of these, 1,971 HCWs underwent only one QFT-GIT test, and 477 HCWs underwent periodic QFT-GIT testing. The initial QFT-GIT results for a total of 2,448 HCWs showed 327 positives (13.4%), 2,118 negatives (86.5%), and three indeterminate (0.1%) results (Table 1). Among them, 1,971 HCWs were tested only once, with 323 positives (16.4%), 1,647 negatives (83.6%), and one indeterminate (0.1%) result. When applying the borderline zone, there were 260 definite positives (13.2%), 63 borderline positives (3.2%), 63 borderline negatives (3.2%), and 1,584 definite negatives (80.4%). Of the 477 HCWs who had more than one QFT-GIT test, their first results included 4 (0.8%) positives, 471 (98.7%) negatives, and 2 (0.4%) indeterminates. When applying the borderline zone, there were 2 definite positives (0.4%), 2 borderline positives (0.4%), 21 borderline negatives (34.4%), and 450 definite negatives (94.3%).

**Table 1:** Baseline QFT-GIT results of healthcare workers with the borderline zone.

Interpretation	Onetime test group		Follow-up test group		Total	
**Definite negative**	1,584	(80.4)	450	(94.3)	2,034	(83.1)
**Borderline negative**	63	(3.2)	21	(4.4)	84	(3.4)
**Borderline positive**	63	(3.2)	2	(0.4)	65	(2.7)
**Definite positive**	260	(13.2)	2	(0.4)	262	(10.7)
**Indeterminate**	1	(0.1)	2	(0.4)	3	(0.1)
**Total**	1,971		477		2,448	

Abbreviation: QFT-GIT, QuantiFERON-TB Gold In-Tube.

### Baseline characteristics of the 477 HCWs with follow-up data

Table 2 summarizes the baseline characteristics of the 477 HCWs who underwent follow-up LTBI screening. They underwent periodic chest X-ray (CXR), and 1,191 QFT-GIT tests at intervals of 1–4 years were performed. The majority of the HCWs were female (88.1%) and were in their twenties (63.3%). Regarding their professions, 41 (8.6%) were doctors, 315 (66.0%) were nurses, 67 (14.0%) were nursing assistants, 26 (5.5%) were medical technicians, 1 (0.2%) was a patient transfer, and 27 (5.7%) were appointee and part-time workers. According to the TB risk classification by workplace, 207 (43.4%) HCWs were at high risk, 222 (46.5%) HCWs were at medium risk and 48 (10.1%) HCWs were at low risk.

**Table 2:** Characteristics of healthcare workers with follow-up QFT-GIT results (n = 477).

Characteristics		n	%
**Sex**	**Male**	57	11.9
	**Female**	420	88.1
**Age**	**20s**	302	63.3
	**30s**	114	23.9
	**40s**	50	10.5
	**50s**	11	2.3
**Profession**	**Doctor**	41	8.6
	**Nurse**	315	66.0
	**Nursing assistant**	67	14.0
	**Medical technician**	26	5.5
	**Patient transfer**	1	0.2
	**Appointee and part-time worker**	27	5.7
**Risk of TB exposure**	**High**	207	43.4
	**Intermediate**	222	46.5
	**Low**	48	10.1

Abbreviation: QFT-GIT, QuantiFERON-TB Gold In-Tube; TB, tuberculosis.

### Serial QFT-GIT results of the 477 HCWs

**Fig 2:**
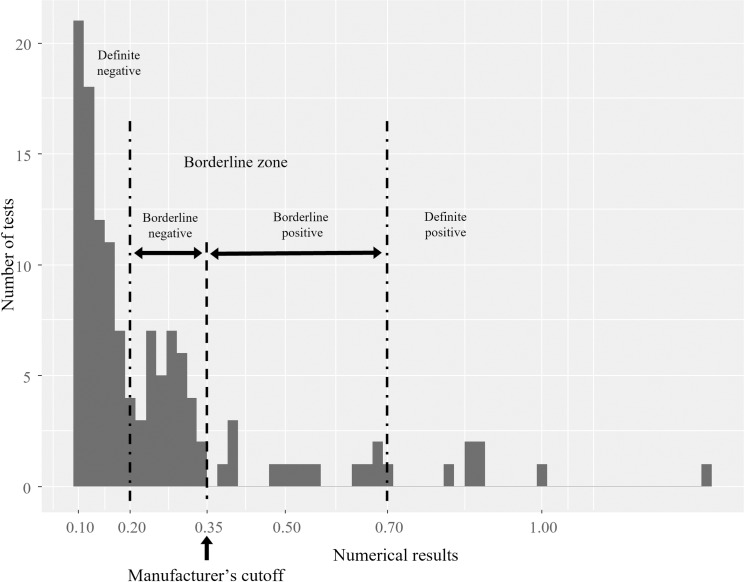
QFT-GIT results (≥ 0.1 to < 1.4 IU/mL) of 477 HCWs with follow-up (n = 128). Abbreviations: QFT-GIT, QuantiFERON-TB Gold In-Tube; HCW, healthcare worker. Among 1,191 results of 477 HCWs, 1,040 results with < 0.1 IU/mL, 18 results with 1.4 > IU/mL and 5 indeterminate results were not shown in the figure.

The interpretation was applied to the 1,191 QFT-GIT results of the 477 HCWs who had follow-up tests (Fig 2). The median number of follow-up tests per HCW was two, with some up to six. The median follow-up interval was 11 months (IQR: 10–12 months). A total of 714 time points was interpreted with the manufacturer’s cutoff compared to previous results. Among them, 676 points (94.7%) were stationary results (results in the same category as the previous test), 30 points (4.2%) were converted, 2 points (0.3%) were reversed, and 6 points (0.8%) could not be analyzed due to indeterminate results (Table 3). When applying the borderline zone, 637 (89.2%) points were stationary results, 22 points (3.1%) were converted, one point (0.1%) was a decision pending, and 6 points (0.8%) could not be analyzed because of indeterminate results. In the order of QFT-GIT tests, the conversion rate with the manufacturer’s cutoff was 3.0% (14/471) for the second test, 5.6% (7/125) for the third, 7.7% (5/65) for the fourth, 6.7% (2/30) for the fifth and 15.4% (2/13) for the sixth. The reversion rate was 25.0% (1/4) for the second test and 50% (1/2) for the third. When applying the borderline zone, the conversion rate was 2.1% (10/471) for the second test, 3.2% (4/125) for the third, 6.2% (4/65) for the fourth, 6.7% (2/30) for the fifth, and 15.4% (2/13) for the sixth. The reversion rate was 0.0% (0/4) for the second test and 50% (1/2) for the third. When analyzing the conversion rate according to the order of the QFT-GIT test by Mann-Kendall trend analysis, the conversion rate (*tau* = 0.8, *P*-value = 0.086) with the manufacturer’s cutoff tended to increase, and the conversion rate of the borderline zone significantly increased (*tau* = 1.0, *P*-value = 0.027).

**Table 3:** Interpretation of serial QFT-GIT results (714 time points, 477 HCWs).

	Manufacturer’s cutoff				Borderline zone			
	By time points		By HCWs		By time points		By HCWs	
**Stationary**	676	(94.7)	441	(92.5)	637	(89.2)	419	(86.8)
**Conversion**	30	(4.2)	30*	(6.3)	22	(3.1)	22*	(4.6)
**Reversion**	2	(0.3)	2*	(0.4)	1	(0.1)	1*	(0.2)
**Decision pending**	NA		NA		48	(6.7)	36	(6.5)
**Indeterminate**	6	(0.8)	5	(1.0)	6	(0.8)	5	(1.0)
**Total**	714		477*		714		477*	

Abbreviations: QFT-GIT, QuantiFERON-TB Gold In-Tube; HCW, healthcare worker; NA, not applicable.

* One HCW showed reversion after conversion.

By individual HCW, the serial QFT-GIT results with the manufacturer’s cutoff for 441 HCWs (92.5%) remained unchanged, including 438 (91.8%) negatives and 3 (0.6%) positives. Thirty HCWs (6.3%) showed conversion, 2 HCWs (0.4%) showed reversion, and a HCW (0.2%) showed reversion after conversion. Five HCWs’ QFT-GIT results (1.0%) could not be interpreted owing to indeterminate results. When applying the borderline zone, the serial QFT-GIT results of 419 HCWs (87.8%) were unchanged, including 414 (86.8%) definite negatives, 2 (0.4%) borderline negatives, 1 (0.2%) borderline positive and 2 (0.4%) definite positives. Twenty-two HCWs (4.6%) showed conversion and a HCW (0.2%) showed reversion. Among two HCWs tested even after conversion, one HCW’s result showed reversion, and the other HCW’s result decreased to borderline positive. During the follow-up period, 36 (7.5%) HCWs remained decision pending, including 5 (1.0%) HCWs with indeterminate results.

### Radiological examination and TB culture results

The clinical features of 31 HCWs with conversion or reversion in QFT-GIT are summarized in S1 Table. A total of 25 of the 31 HCWs visited the outpatient clinics for infectious diseases with their own decision, and 19 HCWs had LDCT. There were one case of active TB in both CXR and LDCT (No. 28) and four cases of active TB in LDCT (No. 2, 18, 21 and 26), despite no specific findings of TB in CXR. In those five HCWs with suspected active TB, TB culture was performed on sputum. Three of them (No. 2, 26 and 28) were positive for *Mycobacterium tuberculosis*. Two HCWs (No. 18 and 21) who were TB culture negative had TB findings only on LDCT. All three HCWs (No. 2, 26 and 28) who were TB culture positive showed TB findings on LDCT, and two HCWs (No. 2 and 28) showed pleural effusion or TB findings on CXR. In addition, there was one HCW (No. 15) whose X-ray showed no specific findings, but LDCT showed granuloma, a sign of past TB infection. A total of 15 HCWs started LTBI treatment, and 12 (80.0%) HCWs completed treatment. Summarizing the outpatient records, a total of 7 HCWs was identified as having TB during follow-up of 477 HCWs. Five TB cases identified during the LTBI screening. One year after LTBI screening, two HCWs were identified as having TB with one culture positive TB (screening QFT-GIT 0.29 IU/mL) and one clinical TB (No. 13, screening QFT-GIT 4.07 IU/mL).

### The maximum difference in QFT-GIT grades during follow-up

During follow-up, the maximum difference in IGRA grades according to the borderline zone was analyzed by individual, except for 5 HCWs with indeterminate results. The grades of the QFT-GIT results of 419 HCWs (87.8%) remained unchanged, including 414 (86.8%) definite negatives, two (0.4%) borderline negatives, one (0.2%) borderline positive and two (0.4%) definite positives. There were 23 HCWs (4.8%) who showed mild changes, with 22 (4.6%) ranging from definite negative to borderline negative and one (0.2%) ranging from borderline negative to borderline positive. Moderate changes were observed in 10 HCWs (2.1%), with eight (1.7%) ranging from definite negative to borderline positive and two (0.4%) ranging from borderline negative to definite positive. Twenty HCWs showed marked changes, from definite negative to definite positive. Among them, seven HCWs had TB, including one in the mild change group, one in the moderate change group, and five in the marked change group (Table 4). In the Cochran-Armitage trend test, the greater the maximum difference in grade, the higher the probability of developing TB (*P*-value <0.001).

**Table 4:** Maximum difference in QFT-GIT grade with borderline zone and the development of tuberculosis (*P*-value <0.001).

Maximum difference in grade	Tuberculosis		No tuberculosis		Total
**No change**	0	(0.0)	419	(100.0)	414
**Mild difference**	1	(4.3)	22	(95.7)	23
**Moderate difference**	1	(10.0)	9	(90.0)	10
**Marked difference**	5	(25.0)	15	(75.0)	20

Abbreviation: QFT-GIT, QuantiFERON-TB Gold In-Tube

## Discussion

In this study, we applied the borderline zone in serial IGRA at a hospital in South Korea with moderate TB incidence. Accordingly, we identified the distribution of the results, the changes in interpretation and the risks of increasing the degree of QFT-GIT.

The positivity rate of the HCWs’ initial IGRA test was 13.4%, which is slightly lower than that of 15.8–23.7% in other South Korean studies [17–19] and between that of 8.3–22.2% in low TB incidence countries [7–9, 11, 20]. Using the manufacturer’s cutoff is simple to interpret but increases conversion and reversion. Overcoming this drawback, applying the borderline zone reveals ambiguous areas [7–12]. With the borderline zone, the conversion rate decreased from 4.5% to 3.1% for a time point and from 6.3% to 4.6% for HCW. This change is higher than that in the low-incidence setting of 2.8% to 1.2% for HCW [9]. The decision pending rate was 6.7% for a time point and 6.5% for HCW. There have been few reports evaluating interpretations based on each time point of follow-up [10], especially ambiguous areas resulting from the introduction of borderline zones.

Reversion was limited to those who were followed up and became negative after being IGRA positive (33.3%; 2/6), which was the same as the existing high reversion rate [5]. It was difficult to evaluate the reversion, as most of the subsequent QFT-GIT tests were not performed after positive results according to the guidelines. Because the number of observed reversions was small, we could not compare the change in TB risk, which remains unclear as that in previous studies [12, 21].

The strategies of introducing the borderline zone were varied by reports [7–9, 11]. They depend on the country’s TB incidence and the characteristics of the hospital where the study was conducted. There are many reports on the dynamics of the QFT-GIT. It is known that the blood collection time, the blood volume, and processing delay can affect the QFT-GIT results and that the between-run reproducibility is poor [6]. Regarding QFT-GIT results in the borderline zone, short-term follow-up showed usefulness [11, 20, 22]. One HCW with borderline negative QFT-GIT results developed TB after one year. Careful observation for HCWs with borderline QFT-GIT results during serial screening is also needed, and it is necessary to determine the treatment by short-term follow-up.

If an HCW has a high risk of TB contact or a risk of transmission, it is recommended to continue LTBI screening and to evaluate their risk. Although the conversion rate is expected to increase with this risk, consistent reports have not been made [5]. We found an increased conversion rate in long-term followers, which was more evident when interpreted with the borderline zone than the manufacturer’s cutoff. Because those who were continuously screened were at high risk, the conversion rate increased as the risk accumulated, but the number of long-term followers was relatively small. The difference between studies may be due to differences in incidence or definition.

It is crucial to exclude active TB during the LTBI screening of HCWs. According to the Korean guidelines for TB and the CDC guidelines for TB, if the LTBI test is positive, a medical examination and CXR should be performed, followed by sputum culture to confirm TB [3, 4]. Although computed tomography (CT) has been reported to be useful in LTBI screenings, it is not yet included in the guidelines, and cost and risk versus effectiveness should be considered [23–28]. LDCT has a lower radiation dose than high-resolution computed tomography (HRCT), which has been shown to be useful in TB contact screening [26, 29]. In this study, we found that it is necessary to rule out the possibility of TB by performing LDCT at the time of LTBI examination as well as CXR for QFT-GIT-positive HCWs. If only CXR and sputum cultures had been used, two cases of clinical TB (40%) identified by LDCT would have been missed. If they had been treated for LTBI, they were mistreated for TB, and refusing LTBI treatment could delay the discovery of TB.

A total of seven HCWs were diagnosed as TB among 477 HCWs. Five HCWs were TB positive at the time of LTBI screening, and the QFT-GIT result was four definite positives and one borderline positive. One year later, two of those identified as having TB on the basis of their QFT-GIT results were definite positive and borderline negative at LTBI screening. However, it was not possible to identify whether the QFT-GIT result was a false negative or whether TB had developed because of new exposure. Those with borderline negative results also need careful examination during follow-up. Combining their previous QFT-GIT results, the greater the maximum difference in grade was, the higher the incidence of TB. This finding is similar to that of a study using a rigorous conversion definition (0.2 to >0.7 IU/mL) to more clearly find recent TB infections [12]. However, the QFT-GIT results in the borderline zone are also TB-specific, indicating TB infection [16]. Therefore, no conclusion about the necessity of LTBI treatment for HCWs with borderline QFT-GIT can be drawn from our data. Regarding those results, a careful approach, including short-term follow-up, is necessary to determine LTBI treatment.

As a retrospective study, it was difficult to obtain information about bacillus Calmette-Guerin (BCG) vaccination, past TB treatment, and history of TB contact from HCWs who did not visit the outpatient clinic. Most of the subsequent QFT-GIT tests were not performed after positive results according to the guidelines. In addition, TB contact history could not be ruled out because the preplacement QFT-GIT was not tested. Short-term follow-up or retesting of QFT-GIT was not performed for the results in the borderline zone. Re-examination would have helped to evaluate a more accurate conversion rate. Since the follow-up period was not long and the possibility of new TB exposure could not be ruled out, it was unclear whether the incidence of TB was due to LTBI activation.

In conclusion, by introducing the borderline zone, we found that the conversion rate decreased, and the decision pending rate increased. For IGRA-positive HCWs, TB was confirmed by LDCT in addition to CXR and sputum culture. The probability of developing TB was associated with the difference in QFT-GIT grade. However, a careful approach is needed to introduce the borderline zone in a moderate TB incidence country. Thus, converters by the borderline zone need a thorough examination to rule out TB, and HCWs with QFT-GIT results in the borderline zone also need close observation, including short-term follow-up.

## Supporting information

S1 File Data file. (XLSX)

S1 Table QFT-GIT and imaging results and treatment among 31 HCWs with positive QFT-GIT. (DOCX)
