# Supplementary material for: The use of a borderline zone for the interpretation of interferon-gamma release assay results for serial screening of healthcare workers
Source: PLoS One. 2020 Jun 30;15(6):e0235254. doi: 10.1371/journal.pone.0235254 (PMC7326217; doi:10.1371/journal.pone.0235254)
Supplement: S1 Table — (DOCX) [file pone.0235254.s002.docx]

**S1 Table. QFT-GIT and imaging results and treatment among 31 HCWs with positive QFT-GIT.**

| No. | Risk | QFT-GIT | | | | | | Interpretation | | CXR | LDCT | Culture | Diagnosis | Treatment | Treatment  Completion |
| --- | --- | --- | --- | --- | --- | --- | --- | --- | --- | --- | --- | --- | --- | --- | --- |
|  |  | 1st | 2nd | 3rd | 4th | 5th | 6th | Manufacturer’s  cutoff | Borderline  zone |  |  |  |  |  |  |
| 1 | High | 0.34 | 0.41 |  |  |  |  | C | P | Not remarkable | NT | NT | Unknown | Unknown | - |
| 2 | High | 0.01 | **4.17** | 0.67 |  |  |  | C | C | Pleural effusion | Active TB | TB | Active TB | TB | Yes |
| 3 | Intermediate | 0.33 | 0.04 | 0.00 | 1.45 |  |  | C | C | Not remarkable | NT | NT | LTBI | LTBI | Yes |
| 4 | High | 0.03 | 1.91 | 0.12 | 0.03 |  |  | C & R | C & R | Not remarkable | NT | NT | Unknown | Unknown | - |
| 5 | High | 0.09 | 1.62 |  |  |  |  | C | C | Not remarkable | NT | NT | LTBI | Refusal | - |
| 6 | High | 0.22 | 0.15 | 0.41 |  |  |  | C | P | Stable TB | NT | NT | Unknown | Unknown | - |
| 7 | Intermediate | 0.15 | 0.18 | 0.29 | 0.70 |  |  | C | C | Not remarkable | NT | NT | LTBI | LTBI | Yes |
| 8 | High | 0.65 | 0.18 |  |  |  |  | R | P | Not remarkable | NT | NT | Unknown | Unknown | - |
| 9 | High | 0.09 | 0.57 |  |  |  |  | C | P | Not remarkable | Not remarkable | NT | LTBI | LTBI | Yes |
| 10 | High | 0.16 | 0.04 | 0.00 | 0.02 | 0.05 | 5.66 | C | C | Not remarkable | Benign nodules | NT | LTBI | LTBI | Yes |
| 11 | High | 0.34 | 0.00 | 0.86 |  |  |  | C | C | Not remarkable | NT | NT | LTBI | Refusal | - |
| 12 | High | 0.00 | 0.00 | 0.00 | 0.02 | 0.86 |  | C | C | Not remarkable | Not remarkable | NT | LTBI | LTBI | No |
| 13 | High | 0.15 | 4.07 |  |  |  |  | C | C | After TB | After TB | Neg | Unknown,  after TB | TB | Yes |
| 14 | High | 0.02 | 0.00 | 1.79 |  |  |  | C | C | Benign granuloma | Benign granuloma | NT | LTBI | LTBI | No |
| 15 | High | 0.00 | 0.06 | 0.49 |  |  |  | C | P | Not remarkable | Benign granuloma | NT | LTBI | LTBI | Yes |
| 16 | High | 0.00 | 0.00 | 0.88 |  |  |  | C | C | Not remarkable | Not remarkable | NT | LTBI | LTBI | Yes |
| 17 | Intermediate | 0.31 | 1.51 |  |  |  |  | C | C | Not remarkable | NT | NT | Unknown | unknown | - |
| 18 | High | 0.03 | 0.02 | 0.01 | 0.20 | **0.83** |  | C | C | Not remarkable | Possible active TB | Neg | Active TB | TB | Yes |
| 19 | High | 0.02 | 0.02 | 0.55 |  |  |  | C | P | Not remarkable | Benign nodules | NT | LTBI | Refusal | - |
| 20 | High | 0.00 | 0.5 |  |  |  |  | C | P | Not remarkable | Not remarkable | NT | LTBI | LTBI | Yes |
| 21 | High | 0.11 | 0.00 | 0.00 | **33.18** |  |  | C | C | Not remarkable | Possible active TB | Neg | Active TB | TB | Yes |
| 22 | High | 0.00 | 0.00 | 2.45 | 0.68 |  |  | C | C | Not remarkable | NT | NT | LTBI | Refusal | - |
| 23 | High | 0.01 | 0.88 |  |  |  |  | C | C | Not remarkable | Bronchiolitis | Neg | LTBI | Refusal → LTBI | Yes |
| 24 | High | 0.01 | 1.65 |  |  |  |  | C | C | Not remarkable | Not remarkable | NT | LTBI | LTBI | Yes |
| 25 | High | 0.04 | 0.38 |  |  |  |  | C | P | Not remarkable | NT | NT | LTBI | LTBI | Yes |
| 26 | High | 0.00 | 0.00 | 0.00 | **0.68** |  |  | C | P | Not remarkable | Active TB | TB | Active TB | TB | Yes |
| 27 | High | 0.00 | 0.08 | 0.25 | 2.10 |  |  | C | C | Not remarkable | Not remarkable | NT | LTBI | Refusal | - |
| 28 | High | 0.00 | 0.03 | **29.59** |  |  |  | C | C | Active TB | Active TB | TB | Active TB | TB | Yes |
| 29 | High | 0.00 | 4.87 |  |  |  |  | C | C | Not remarkable | Benign nodules | NT | LTBI | LTBI | Yes |
| 30 | High | 0.00 | 0.03 | 0.00 | 0.00 | 0.02 | 1.33 | C | C | Not remarkable | Not remarkable | NT | LTBI | LTBI | Yes |
| 31 | High | 0.00 | 2.42 |  |  |  |  | C | C | Not remarkable | Not remarkable | NT | LTBI | LTBI | No |

Abbreviations: QuantiFERON-TB Gold In-Tube test, QFT-GIT; healthcare worker, HCW; chest X-ray, CXR; low-dose chest CT, LDCT; conversion, C; reversion, R; decision pending, P; not tested, NT; tuberculosis, TB; latent tuberculosis infection, LTBI.

Bold text: QFT-GIT results at the time point of the diagnosis of tuberculosis.
